# Passive radio frequency identification and video tracking for the determination of location and movement of broilers

**DOI:** 10.1016/j.psj.2022.102412

**Published:** 2022-12-09

**Authors:** J.E. Doornweerd, G. Kootstra, R.F. Veerkamp, B. de Klerk, I. Fodor, M. van der Sluis, A.C. Bouwman, E.D. Ellen

**Affiliations:** ⁎ Animal Breeding and Genomics, Wageningen University & Research, 6700 AH Wageningen, the Netherlands; † Farm Technology, Wageningen University & Research, 6700 AA Wageningen, the Netherlands; ‡ Research & Development, Cobb Europe BV, 5831 GH Boxmeer, the Netherlands

**Keywords:** broiler, activity, deep learning, tracking, sensors

## Abstract

Phenotypes on individual animals are required for breeding programs to be able to select for traits. However, phenotyping individual animals can be difficult and time-consuming, especially for traits related to health, welfare, and performance. Individual broiler behavior could serve as a proxy for these traits when recorded automatically and reliably on many animals. Sensors could record individual broiler behavior, yet different sensors can differ in their assessment. In this study a comparison was made between a passive radio frequency identification (**RFID**) system (grid of antennas underneath the pen) and video tracking for the determination of location and movement of 3 color-marked broilers at d 18. Furthermore, a systems comparison of derived behavioral metrics such as space usage, locomotion activity and apparent feeding and drinking behavior was made. Color-marked broilers simplified the computer vision task for YOLOv5 to detect, track, and identify the animals. Animal locations derived from the RFID-system and based on video were largely in agreement. Most location differences (77.5%) were within the mean radius of the antennas’ enclosing circle (≤128 px, 28.15 cm), and 95.3% of the differences were within a one antenna difference (≤256 px, 56.30 cm). Animal movement was not always registered by the RFID-system whereas video was sensitive to detection noise and the animal's behavior (e.g., pecking). The method used to determine location and the systems’ sensitivities to movement led to differences in behavioral metrics. Behavioral metrics derived from video are likely more accurate than RFID-system derived behavioral metrics. However, at present, only the RFID-system can provide individual identification for non-color marked broilers. A combination of verifiable and detailed video with the unique identification of RFID could make it possible to identify, describe, and quantify a wide range of individual broiler behaviors.

## INTRODUCTION

Breeding programmes require phenotypes on individual animals to be able to select for traits. However, not every novel trait for the breeding goal is easy to measure. The assessment of traits related to health, welfare, and performance can be time-consuming, difficult, or both. Alternatively, individual broiler behavior could serve as a proxy for an individual's health, welfare, and performance (Hart, 1988; Tickle et al., 2018; van Hertem et al., 2018). Furthermore, understanding individual broiler behavior could provide insight into animal social interactions and pen dynamics. However, individual broiler behavior will need to be recorded reliably and automatically on many animals to be implemented in practice.

Several sensors for recording individual broiler behavior, both wearable and remote, have been introduced over the years. Wearable sensors include, for example, accelerometers (Yang et al., 2021), inertial measurement units (**IMUs**; Derakhshani et al., 2022), radio frequency identification (**RFID**; van der Sluis et al., 2020) and ultra-wideband tracking (**UWB**; van der Sluis et al., 2019). Wearable sensors could be considered impractical and expensive, as all animals need a sensor for individual phenotypes, and some sensors need batteries (accelerometers, IMU, UWB) or an extensive network of antennas (RFID). However, advances in battery operated wearable sensors (accelerometers, IMU, UWB) will improve battery lifetime and reduce costs in the future. In contrast to wearable sensors, there are remote sensors, such as microphones (Huang et al., 2019), 2D cameras (van Hertem et al., 2018), 3D cameras (Matthews et al., 2017), and infrared thermography cameras (Zaninelli et al., 2016). A single remote sensor, such as a video camera, could monitor multiple animals, but individual identification is challenging, and the collected data is high-dimensional spatial data from which the desired information needs to be extracted. The challenge with remote sensors, and more specifically video cameras, is to detect, identify and track the animals in commercial environments under varying conditions (e.g., group size, stocking density, lighting, background). Despite the introduction of numerous sensors in research, neither wearable nor remote sensors are widely used in practice in broilers.

Both wearable and remote sensors could provide individual broiler behavioral data. The sensors may be used to identify animals, determine their location, detect activity, provide detailed behavioral data, or to provide a combination of these features. In turn, behavioral metrics could be derived from these features (e.g., space usage, locomotion activity, feeding and drinking behavior). In broilers, locomotion activity has been indicated as a potential proxy for an individual's health, welfare, and performance (Hart, 1988; Tickle et al., 2018; van Hertem et al., 2018) and may thus be interesting to record. Measuring locomotion activity requires sensors that can track an animal's location and movement over time.

The objective of this study is to compare RFID and video for the determination of location and movement in broilers. We show the potential of RFID and video to assess individual behavioral metrics, such as space usage, locomotion activity and apparent feeding and drinking behavior, derived from both systems under the same circumstances. To show the potential of video, and forgo previously mentioned challenges regarding detecting, identifying, and tracking, we use color-marked broilers to simplify the computer vision task.

## MATERIAL AND METHODS

### Data Acquisition

#### Animals and Housing

The experimental data used in this study have previously been described in the research of van der Sluis et al. (2020) and Doornweerd et al. (2022). Forty male broilers from 2 genetic crosses were housed in one pen (1.80 × 2.61 m). At hatch, all birds were fitted with an RFID-tag. Four birds, 2 from each cross, were marked in 4 different colors, 3 of which were used in this study (black, light blue, and pink), because one bird's color (dark blue) was inconsistent over days. From 15 d of age, 34 out of 40 birds were equipped with an UWB-tag, including the color-marked birds. The UWB data were not used in this study, but the UWB-tag could shift forward and affect the bird's movement. In the present study, only RFID and video data at 18 d of age were used. The birds were kept under standard lighting conditions. Four feeders and one drinker provided feed and water ad libitum (van der Sluis et al., 2020).

#### Radio Frequency Identification System

**Figure 1:**
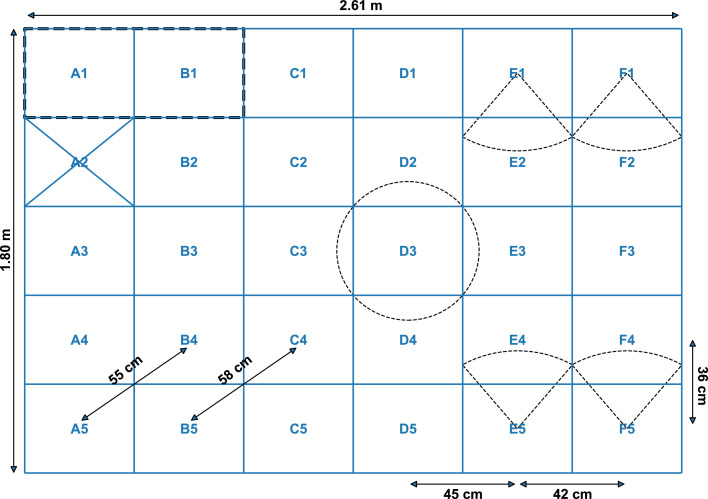
Schematic top view of the radio frequency identification (RFID)-grid. One of the fifteen PVC panels is outlined. Distances between antenna center points are shown. The defective antenna (A2) is crossed out. The feeders were positioned on antennas E1, F1, E5 and F5. The drinker was positioned on antenna D1. An antenna's enclosing circle is illustrated at antenna D3. Circular sectors are indicated for the feeders. Reproduced and adapted with permission from van der Sluis et al. (2020).

A passive RFID-system (Dorset Identification B.V., Aalten, the Netherlands) was used to track individual broilers. Each broiler was fitted with a high-frequency RFID-tag (13.56 MHz, 15 × 3.7 mm, <1.0 gm) around its leg, attached using a rubber band and a piece of tape (van der Sluis et al., 2020, see Figure 4.1). In total, 30 subfloor high-frequency RFID-antennas (32 × 41 cm each) covered the entire surface area of the pen. The antennas were mounted on 15 PVC panels in pairs of two antennas adjoined at the short side. Distance between center points of the antennas of the RFID-grid differed depending on the antenna position in relation to the PVC panels. Antenna A2 was defective during this trial (Figure 1). The RFID-system continuously recorded time, RFID-tag, and the antenna that registered the tag using 2 readers (HF RFID reader DSLR1000, freaquent froschelectronics GmbH, Graz, Austria) running on custom software from the same company (van der Sluis et al., 2020).

#### Video

An RGB-camera placed above the pen recorded the birds from 10:44 am to 12:59 pm at 18 d of age. The 2-h period was recorded in a series of 20 videos of approximately 7 minutes each. The videos were recorded in full-HD (1920 × 1080 px) at 25 frames per second with a Zavio B6210 2MP camera (Zavio Inc., Hsinchu City, Taiwan). Video was used for human annotation of ‘ground-truth’ to train the computer vision detection model described below.

#### Frame Extraction and Annotation

Five-hundred frames were randomly selected from all extracted video frames for ‘ground-truth’ annotation of the 3 color-marked birds. The selected frames were randomly divided between 2 human annotators (annotator A and B). The Computer Vision Annotation Tool (CVAT; Sekachev et al., 2019) was used for annotation. The annotators annotated the selected frames by manually drawing bounding boxes around the three color-marked birds, each bird with a specific color-based class (black, light blue, and pink).

Annotation quality and agreement between annotators was assessed with the intra- and inter-annotator reliability. To calculate intra- and inter-annotator reliability, both annotators annotated the same 25 frames, randomly selected from the 500 frames, twice. Reliability was assessed with the intersection over union (**IOU**), calculated by dividing the area of overlap (intersection) with the area of the union of the respective bounding boxes (Equation (1)).

(1) $$IOU\left(A,\;B\right)=\frac{\left|A\cap B\right|}{\left|A\cup B\right|}$$

Where A is the bounding box from annotator A, and B the bounding box from annotator B for inter-annotator reliability, or A and B representing bounding boxes from the same annotator annotated at different points in time for intra-annotator reliability.

The quality of annotation and agreement between annotations were considered sufficient to continue model training. Intra-annotator reliabilities were 0.95 (interquartile range [**IQR**] = 0.04) and 0.95 (IQR = 0.07) for annotator A and B, respectively, and the inter-annotator reliability was 0.92 (IQR = 0.08), expressed in median IOU.

### RFID-based Localization and Distance Measurements

The RFID-system localized the birds by reads of the RFID-tags on the antenna grid as an antenna number. In the absence of a new read, the RFID-system continued to report the antenna number of the last read for 5 seconds. In case of double reads, that is, two reads within one second, the first received antenna number was retained. The center point of the recorded antenna was used as animal location. In case of missing reads the birds were assumed to have remained at the last detected location. Distance measurements (distance moved) were calculated as the Euclidean distance in meters between center points of the recorded antennas between consecutive locations in time (Equation (2); Figure 1; van der Sluis et al., 2020).

(2) $$d=∥x-y{∥}_{2}$$

Where x and y are consecutive locations over time.

### Video-based Localization and Distance Measurements

#### Deep Neural Network for Broiler Detection

**Figure 2:**
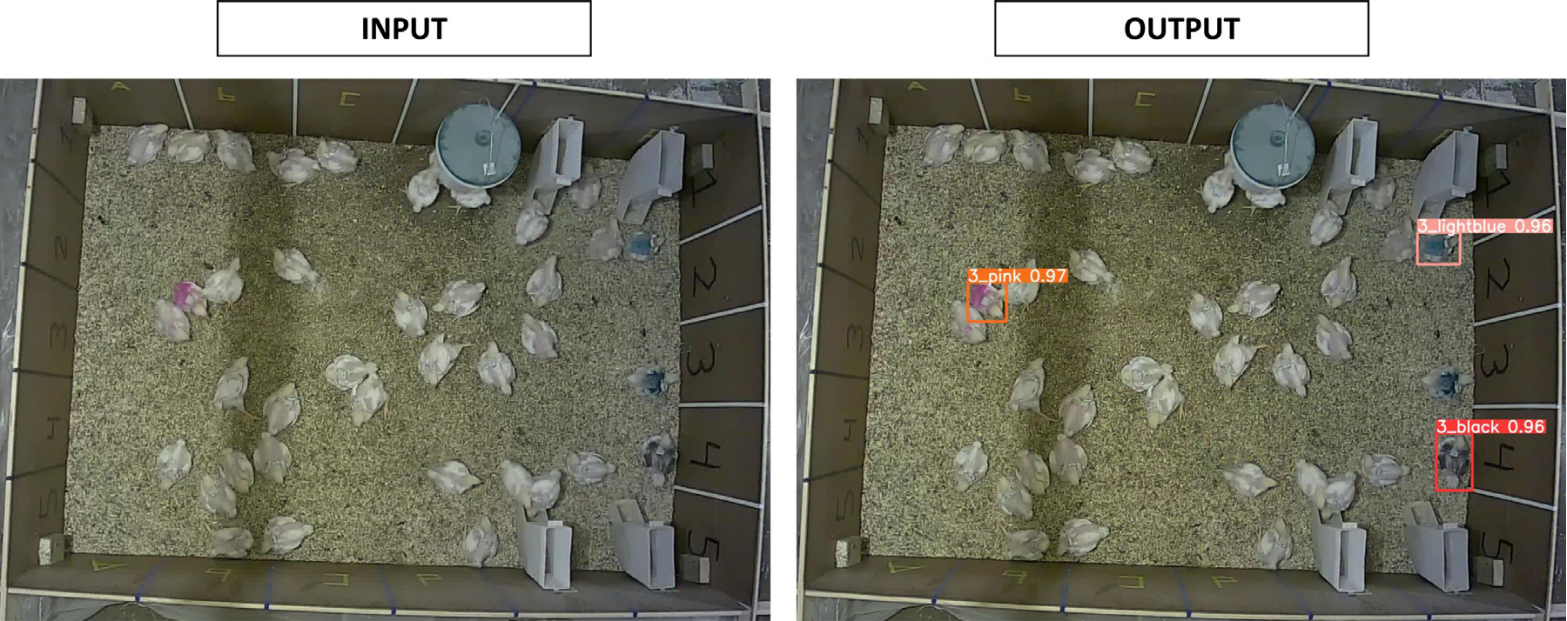
Input and output YOLOv5s. YOLOv5s returns bounding boxes and classifications for the objects of interests, in this case three color-marked broilers. See YOLOv5s repository for detailed architecture (Jocher et al., 2021).

A deep neural network called YOLOv5s (Jocher et al., 2021) was used to detect the color-marked birds on video. YOLOv5s takes an input image and predicts bounding box coordinates, classes, and confidence scores for objects of interest (Figure 2). YOLOv5s is a single-stage object detector, consisting of three components, a backbone for feature extraction (CSPDarknet53), a neck to fuse features (PANet), and a head for bounding box and class predictions (YOLO). The output of YOLOv5s is a series of object predictions, where each prediction included the predicted class of the object (bird color in this work), its bounding box coordinates (center point, width, and height), and the confidence score indicating how certain the model was about the prediction. A confidence threshold was used to filter the prediction based on their confidence score. The value of the threshold depended on the evaluation performed, as described in more detail later. The YOLOv5s model was pretrained on the Microsoft Common Objects in Context (**COCO**) dataset (Lin et al., 2014). The pretrained YOLOv5s-model was trained, validated, and tested using the 500 annotated frames. The frames (n = 500) were randomly split into a train (n = 350 frames), a validation (n = 75 frames), and a test set (n = 75 frames). The model was trained with mostly default hyperparameters, except for batch size (lowered to 6; Supplementary data).

#### Localization and Distance Measurements

On video, animal location was based on the center point coordinates of the bounding boxes detected by YOLOv5s. The detections were reduced from 25 frames per second to 1 frame per second to match the RFID-system. Per class, the detection with the highest confidence within the second was retained, unless more than 80% of the frames within the second had no detection in which case a missing detection was reported. It was observed that detection switched between the light blue color-marked broiler and the dark blue color-marked broiler if the light blue color-marked broiler was occluded. To minimize the impact of the switches on the determination of location the IOU (Equation (1)) between detections within a class was calculated. Two consecutive nonoverlapping detections signified a potential switch, in which case the animal was assumed to have remained at its previous location. In case of missing detections, the birds were assumed to have remained at the last detected location.

Distance measurements were calculated as the Euclidean distance in pixels between consecutive locations over time (Equation (2)). Distance measurements were converted from pixels to meters with a conversion factor derived from the known length of one side of the pen. In the absence of camera calibration and the oblique viewing angle, this conversion resulted in an approximated distance (we call this “pixel-based”).

### Performance Evaluation

#### Evaluation of Video-based Broiler Detection

Different metrics were used to evaluate YOLOv5s’ ability to detect color-marked broilers on video. The different evaluation metrics detailed below were based on the number of true positives (**TP**), false positives (**FP**) and false negatives (**FN**) between predictions and ground-truth annotations. Whether a prediction was a TP, FP or FN depended on the confidence score, classification, and IOU with the ground-truth annotation. A prediction was a TP if the confidence score was greater than or equal to the confidence threshold, had a correct classification, and the IOU was greater than or equal to the IOU-threshold. A FP was a prediction with a confidence score greater than or equal to the confidence threshold, but with either an IOU greater or equal to the IOU-threshold but with an incorrect classification (wrong class), or an IOU less than the IOU-threshold (wrong spatial coordinates). A FN was a broiler that was not matched with a prediction, that is, when there was no prediction of the right class and with the IOU greater or equal to the IOU-threshold.

Precision (proportion of correct predictions; Equation (3)) and recall (proportion of detected broilers; Equation (4)) depend on the selected confidence threshold, and they are calculated by:

(3) $$p=\frac{TP}{TP+FP}$$

(4) $$r=\frac{TP}{TP+FN}$$

A precision-recall curve was constructed by varying the confidence threshold and determining the precision and recall per confidence threshold for a given IOU-threshold. The average precision (**AP**; Equation (5)) is a metric that summarizes the precision-recall curve per class using an interpolation of the precision-recall curve and summing up the interpolated precision values for *N* different equally spaced recall values. We calculated AP according to the standard used in COCO (COCO Detection Challenge, 2019):

(5) $$AP=\frac{1}{N}\sum_{r\in {\mathbb{R}}_{N}}p_{interp}\left(r\right)\;\;p_{\text{interp}}\left(r\right)=\max_{r^{'}:r^{'}\;\geq \;r}p\left(r^{'}\right)$$

Where $R_{N}$ is a set of N=101 uniformly distributed recall values from 0 to 1 and $\;p_{\text{interp}}\left(r\right)$ denotes the interpolated precision at the reference recall value r.

The mean average precision (**mAP**; Equation (6)) is the AP over multiple classes. The mAP can be calculated for single and multiple IOU thresholds, for example, mAP@[.5:.05:.95] is the average mAP at 10 different IOU-thresholds ranging from 0.5 to 0.95 with steps of 0.05.

(6) $$\text{mAP}=\;\frac{1}{C}\sum_{i=1}^{C}AP_{i}$$

Where $AP_{i}$ is the AP for the *i*th class and C is the total number of classes (C = 3).

Precision and recall are reported for the confidence threshold that maximized F1 (Equation (7)) and an IOU-threshold of 0.5. The F1-score is the harmonic mean of precision and recall:

(7) $$F1=\;2*\frac{p*r}{p+r}$$

All metrics have a value between 0 and 1 in which 1 is perfect.

#### Localization Agreement

A comparison between the RFID-system derived and video-based animal locations was made to assess the localization agreement between both systems. This comparison was performed using two different approaches, one based on Euclidean distances and the other on total time spent per antenna differences. These two approaches help to deduce the difference between both systems in determining animal's location and movement over time.

In the first approach, differences in RFID-system derived and video-based animal locations were assessed with the Euclidean distance. The Euclidean distances between corresponding locations in time from both systems were calculated using Equation (2), with x and y as locations estimated by the two different systems at the same timepoint. The localization agreement was evaluated as the percentage of differences less than or equal to the mean radius of the antennas’ enclosing circle (≤128 px, 28.15 cm). The mean radius of the antennas’ enclosing circle encompasses a single value, opposed to the length and width of the antennas. Furthermore, this method of evaluating localization agreement accounts for the fact that the RFID-system was restricted to movement between antennas, whereas video could capture movement both within and between antennas. For the localization agreement, video-based locations that appeared on the defective antenna A2 were excluded.

In the second approach, differences in RFID-system derived and video-based animal locations were assessed with regards to total time spent per antenna in seconds. To make a more direct comparison between RFID-system derived and video-based animal locations, video locations were made to mimic the RFID-system. The center point coordinates of the detected bounding boxes by YOLOv5s were translated to RFID-antennas (i.e., a “video-as-RFID” scenario). In case the center point of the bounding box was outside the predefined antenna grid, which happened when the birds were close to the wall of the pen, the detection was considered missing. Thus, the birds were assumed to have remained at the last detected location.

### Behavioral Metrics

Behavioral metrics were assessed to show the potential of an RFID-system and video to assess individual behavioral metrics. In this study, the behavioral metrics space usage, locomotion activity and apparent feeding and drinking behavior were measured and compared between both systems. The defective antenna had a minimal effect on locomotion activity and other behavioral metrics (Appendix Tables A-1 and A-2). The four behavioral metrics are described below.

#### Space Usage

For both systems, space usage was defined as the total time spent per unique antenna in seconds. In this case, video-as-RFID was used to generate an antenna grid based heatmap. Furthermore, the number of unique visited antennas and the total number of switches between antennas was calculated. Space usage has overlap with the localization agreement because similar locations will result in similar approximations of space usage.

#### Locomotion Activity

Locomotion activity, expressed as distance moved was calculated for the RFID-system as the sum of the Euclidean distances between the center points of the recorded antennas of the RFID-grid in meters. For the evaluation of the camera-based system, it was calculated as the sum of the converted Euclidean distances between the center point coordinates of the bounding boxes detected by YOLOv5s in meters (pixel-based). The pixel-based total distance moved was calculated for 3 different minimum movement thresholds ($\tau_{m}\in \left\{0,\;0.005,\;0.05\right\}\;\left[m\right]$; Equation (8)). These different minimum movement thresholds were used to examine the effect of filtering out small movements on the total distance moved. The pixel-based distance, $D^{cam}$, was then calculated as:

(8) $$D^{cam}=\;\sum_{t=1}^{T}d_{t}^{cam}*I\left(d_{t}^{cam}\;>\;\tau_{m}\right)$$

Where $d_{t}^{cam\;}=\;∥x_{t}^{cam}-x_{t-1}^{cam}{∥}_{2}$ is the Euclidean distance between 2 consecutive locations x over time (t), and I($d_{t}^{cam}\;>\;\tau_{m}$) is 1 if $d_{t}^{cam}\;>\;\tau_{m}$ is true (distance moved above the minimum movement threshold) and 0 if $d_{t}^{cam}\;>\;\tau_{m}$ is false.

Furthermore, video-as-RFID distances were calculated with the same method as described for the RFID-system. In order to make a more direct comparison between RFID-system derived and video-based locomotion activity.

#### Apparent Feeding and Drinking Behavior

The apparent feeding and drinking behavior were measured based on the animal's location in relation to the feeders (antennas E1, F1, E5, and F5) and drinker (antenna D1). Feeding and drinking behavior were recorded as the total number of visits and the total duration of those visits in seconds. Feeding and drinking behavior were apparent because an animal's presence on a feeder or drinker associated RFID-antenna does not equate actual feeding or drinking behavior.

The apparent feeding behavior on video was determined as pixel-based and as video-as-RFID. To determine pixel-based apparent feeding behavior, the presence of the animal's bounding box center point within a circular sector ($\theta :120^{\circ },\;r:100\;px\;\text{equal}\;\text{to}\;21.99\;\text{cm})$ centered on the midpoint of the feeder opening was used (Figure 1). Apparent drinking behavior on video was only determined as video-as-RFID, because the animal had to move on the antenna to drink. Apparent feeding and drinking behavior derived from the RFID-system were determined from the reads of the RFID-system on the RFID-antenna associated with a feeder or the drinker. No filter was applied on the apparent feeding and drinking behavior.

## RESULTS

### Annotation and Detection Using YOLOv5s

The ability of YOLOv5s to detect color-marked broilers on video, that is, its detection performance, is shown in Table 1 as precision, recall, and mAP. YOLOv5s classified all detected color-marked broilers correctly, but sporadically missed a color-marked broiler. YOLOv5s achieved an mAP@0.5 of 0.99 and an mAP@[.5:.05:.95] of 0.80. YOLOv5s performs well at an IOU-threshold of 0.5, although a stricter IOU-threshold lowered the mAP. This demonstrates that YOLOv5s can detect color-marked broilers on video.

**Table 1:** Detection performance of YOLOv5s on train, validation, and test set.

Metric	Train	Validation	Test
Precision	1	0.99	1
Recall	0.99	0.98	0.97
mAP@0.5	0.99	0.99	0.99
mAP@[.5:.05:.95]	0.95	0.81	0.80

Mean average precision (mAP) at different intersection over union thresholds (@[0.5] or @[.5:.05:.95]).

### Localization Agreement

**Figure 3:**
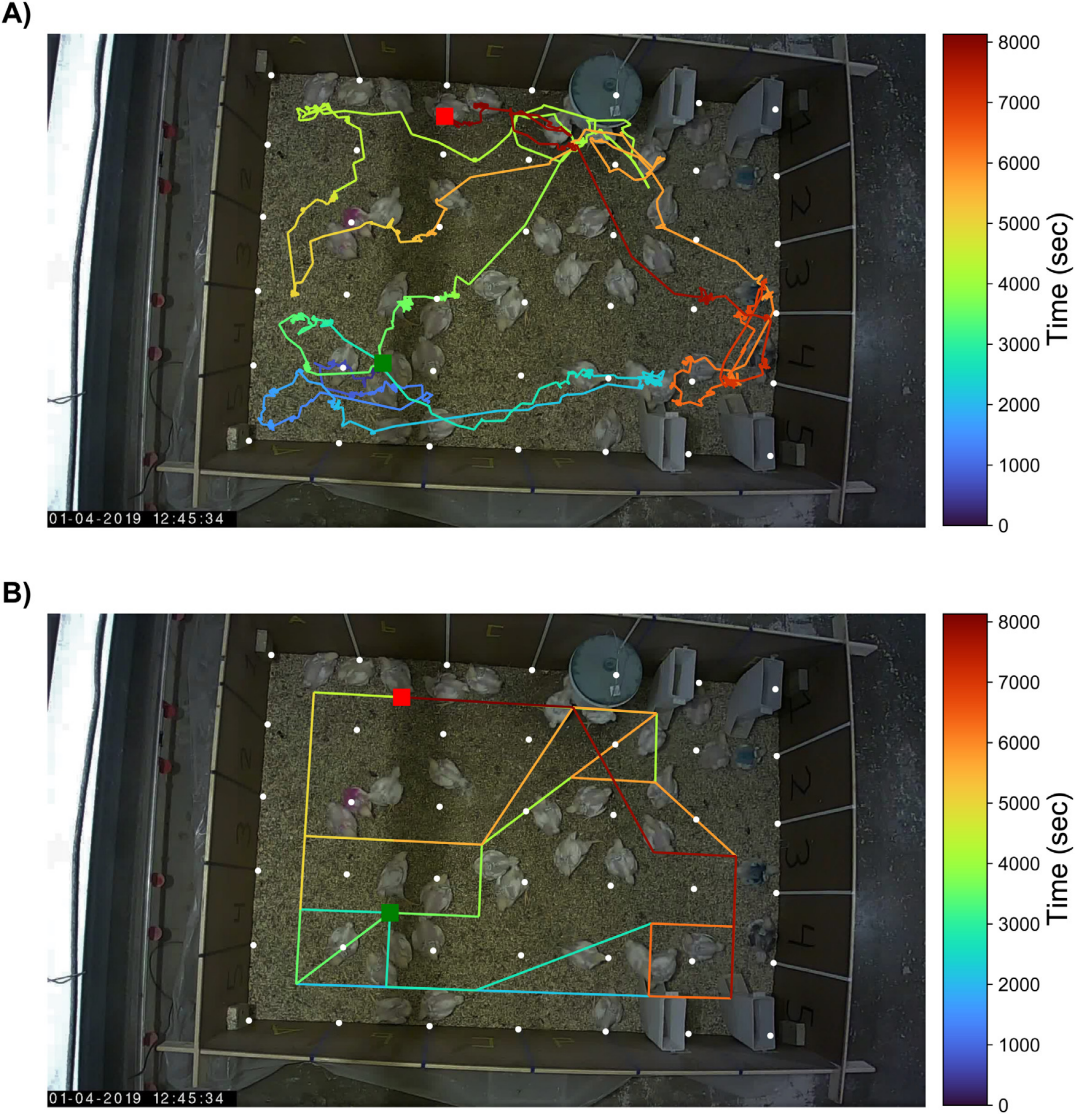
Location of the black color-marked broiler over time as derived from video (A) and radio frequency identification (RFID; B). The green square represents the track's start, and the red square represents the track's end. The color transition depicts the location's progression from dark blue to dark red over time. The white dots represent the antenna corners. The plot is projected over a darkened video freeze frame.

**Figure A-1:**
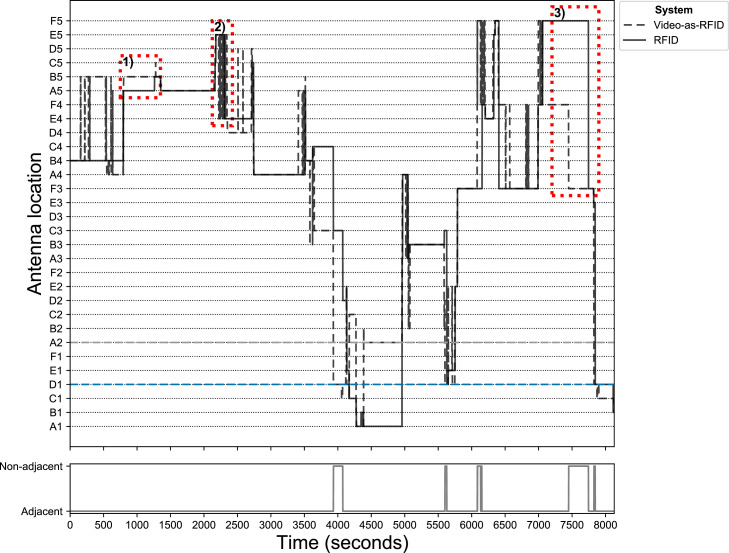
Antenna location of the black color-marked broiler over time as derived from radio frequency identification (RFID; solid line) and video-as-RFID (dashed line). Highlighted with red boxes are 3 events; 1) nonregistered small movement, 2) antenna switches caused by small movements of bounding box's center point, 3) movement to nonadjacent antenna. Highlighted by horizontal dashed lines are the defective antenna (A2; gray dashed line) and drinker antenna (D1; blue dashed line). Nonadjacent antenna location differences between RFID and video shown in bottom plot.

The localization agreement was based on the RFID-system derived and the video-based trajectories of the birds. An example of the RFID-system derived, and video-based locations of the black color-marked broiler is shown in Figure 3. The trajectories started on the same antenna (B4), but on video the track ended on the edge of two antennas. The RFID-system read the RFID-tag on the broiler's leg on antenna B1. The RFID-system was restricted to movement between antennas, whereas video could capture movement both within and between antennas. Periods of minimal movement are shown in the video-based trajectory as an accumulation of points near each other, for instance, those in front of the feeders. Occasionally, movement between antennas could go undetected by the RFID-system, for example, the movement after the bird's first feeder visit in the direction of the starting point. Logically, the video-based trajectory provided more detail than the trajectory derived from the RFID-system. Similar observations could be made for the other color-marked birds (Supplementary data). A detailed overview of antenna locations, both from RFID and from video-as-RFID, of the black color-marked broiler over time can be found in Appendix Figure A-1.

**Figure 4:**
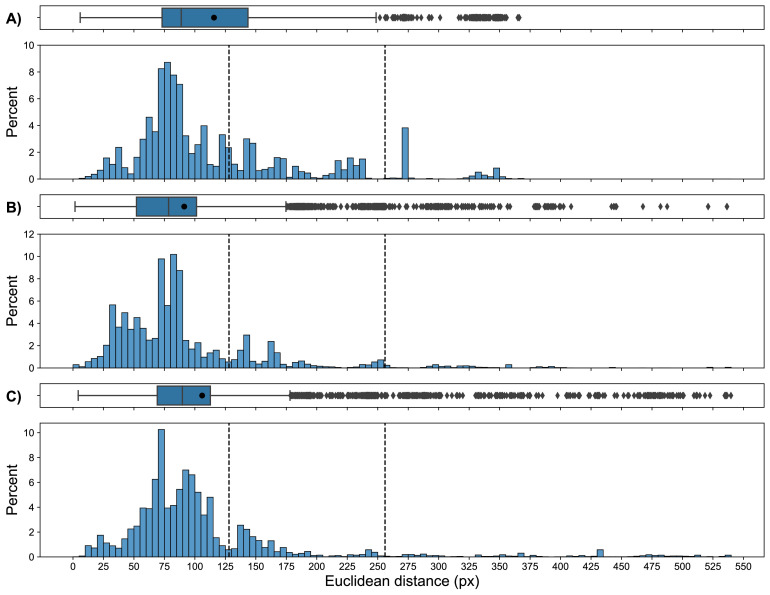
Differences in location expressed as Euclidean distance between locations as derived from radio frequency identification (RFID) and video. Differences in location are shown for A) the black color-marked broiler, B) the light blue color-marked broiler, and C) the pink color-marked broiler. Locations on video within antenna A2 were excluded. The vertical dashed lines indicate the mean radius of the antennas’ enclosing circle (128 px) and a multiple (256 px). Values to the right of the first vertical dashed line signify a difference that crosses antennas, that is, a broiler is assumed to be on another antenna than its current position on video. The black circle in the boxplot shows the mean difference.

The RFID-system derived and video-based animal locations agreed most of the time (Figure 4). Differences, expressed as Euclidean distances between corresponding locations in time, were within the mean radius of the antennas’ enclosing circle (≤128 px, 28.15 cm) 77.5% of the time. Differences greater than the mean radius of the enclosing circle largely occurred within a one antenna difference (≤256 px, 56.30 cm) and accounted for 95.3% of all differences. Occasionally, differences in locations of at least two antennas (≥384 px, 84.45 cm) were observed, particularly for the pink color-marked broiler and to a lesser extent for the light blue color-marked broiler. The mean Euclidean distances between the systems within the trajectories of black, light blue, and pink color-marked broiler were 115.7 px (25.4 cm), 91.2 px (20.1 cm), and 105.9 px (23.3 cm), respectively.

**Figure 5:**
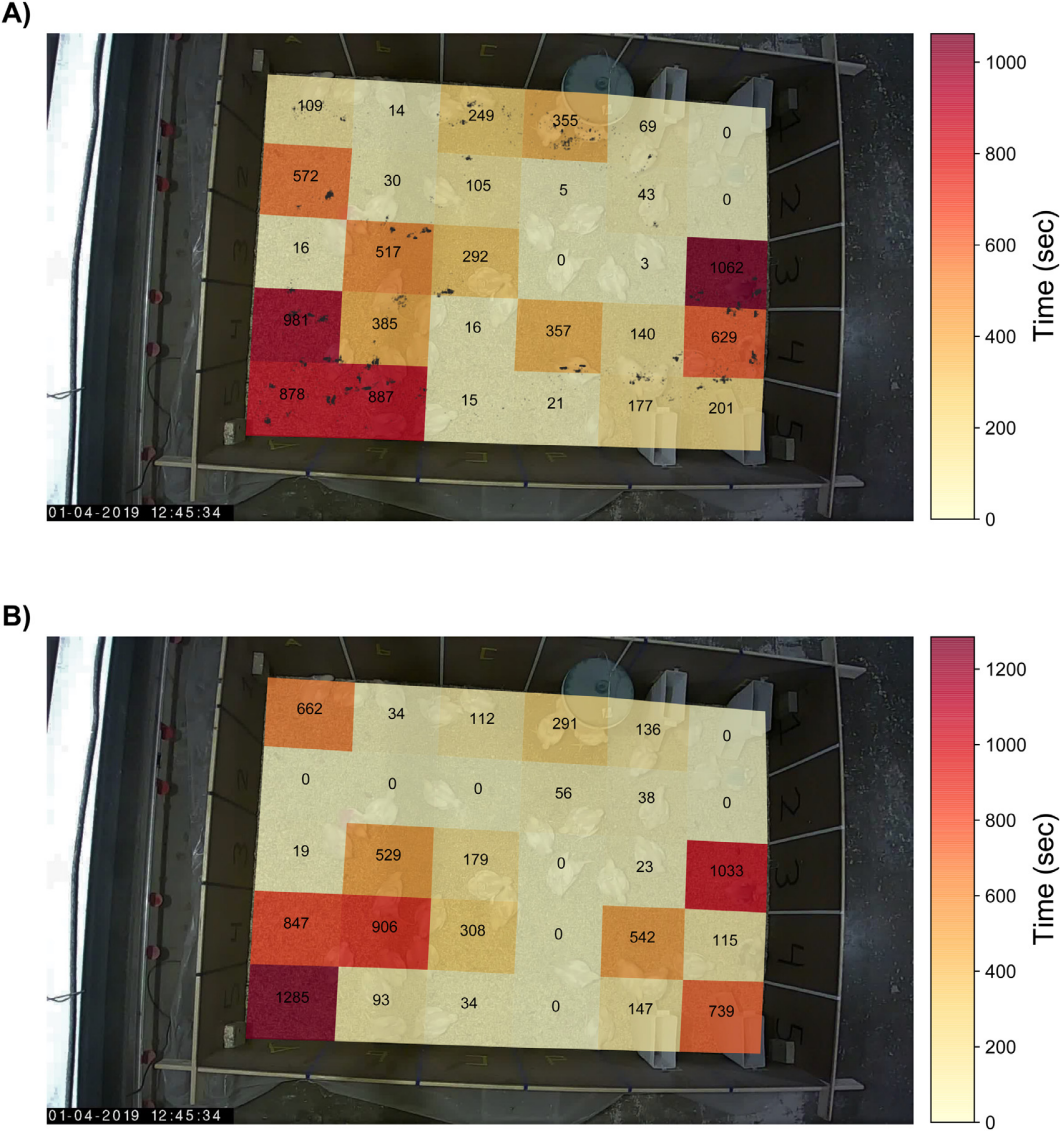
Heatmaps of the black color-marked broiler as derived from video (A) and radio frequency identification (RFID; B). The numbers represent the total amount of time spent on each antenna in seconds. RFID antenna A2 was defective. The animal's bounding box center point locations are included in the heatmap derived from video locations (A) as black dots. The plot is projected over a darkened video freeze frame.

**Figure A-2:**
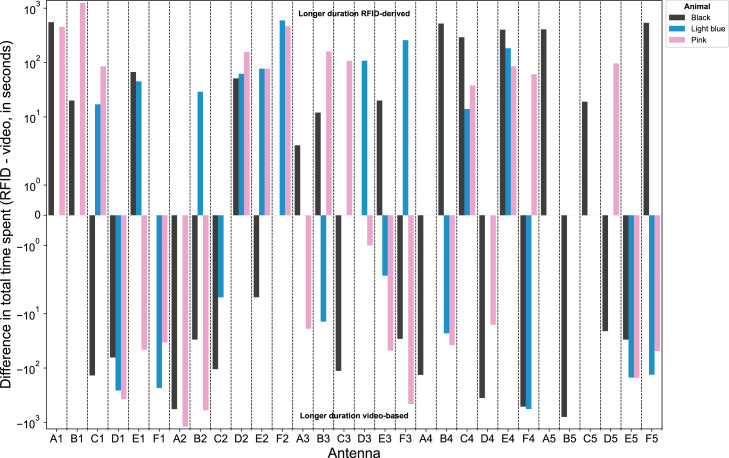
Difference between radio frequency identification (RFID) and video-as-RFID in total time spent per antenna per color-marked broiler on a symmetric log-scale.

Differences in RFID-system derived and video-based animal locations were also assessed with regards to total time spent per antenna in seconds (Table 2). An example heatmap of total time spent per antenna of the RFID-system derived and video-based locations of the black color-marked broiler is shown in Figure 5. This heatmap illustrates the agreement between the RFID-system and video. The RFID-system and video both showed a similar general area pattern of total time spent per antenna. However, the total time spent per individual antenna differed between the RFID-derived and video-based animal locations (Table 2). Similar observations were made for the other color-marked birds (Appendix Figure A-2, Supplementary data).

**Table 2:** Number of unique visited antennas, difference total time spent per antenna between radio frequency identification (RFID) and video (mean ± SD), and number of switches between antennas as derived from RFID and video-as-RFID including defective antenna.

	Number of unique visited antennas (#)		Difference total time spent per antenna (sec)	Number of switches between antennas (#)	
Broiler	RFID	Video-as-RFID		RFID	Video-as-RFID
Black	22	27	0.00 ± 302.15	47	196
Light blue	16	17	0.00 ± 179.13	52	157
Pink	21	26	0.00 ± 377.43	55	233

The largest difference in total time spent on an antenna between RFID and video was found for the pink color-marked broiler with a duration of 20.92 min longer for the RFID-system compared to video. However, this difference can be attributed to the defective antenna. The black and the light blue color-marked broilers had differences of up to +13.23 min on video and +9.90 min for the RFID-system, respectively. The total number of unique antennas visited, and the number of antenna switches was lower for the RFID-system than for video (Table 2). The number of antennas visited differed up to 5 antennas between the RFID-system and video. The RFID-system reported 51 antenna switches and video-as-RFID 195 antenna switches on average across the 3 color-marked birds over the 2 h of recording (Table 2).

The presence on or movement over an antenna for the broilers was not always registered, that is, movement was registered that was not to an adjacent antenna. Ten of these events could be identified for the black and pink color-marked broiler each, and 6 for the light blue color-marked broiler when using the RFID-system. On video, 5 of these events were identified for the light blue color-marked broiler.

### Behavioral Metrics

#### Locomotion Activity

Overall, locomotion activity, defined as the total distance moved in meters, differed substantially between the RFID-system derived and video-based locomotion activity (Table 3, Appendix Table A-2). Compared to video-based locomotion activity, the RFID-system appeared to underestimate the total distance moved. The largest difference was observed for the pink color-marked broiler with a difference of 66.57 m between RFID and video-as-RFID. The use of minimum movement thresholds in video pixel-based activity lowered the difference between video and RFID. The largest within-broiler difference between RFID and video pixel-based distance moved decreased from 54.86 m to 42.03 m (threshold: 5 mm) and 10.70 meters (threshold: 5 cm), respectively for the pink color-marked broiler. This showed that small movements of the center point of the detected bounding boxes by YOLOv5s accumulated to a substantial part of the total distance moved. Differences between animals were more pronounced in video-based locomotion activity than in RFID-derived locomotion activity. The largest difference between animals in RFID-derived locomotion activity was 3.75 m (black vs. pink color-marked broiler), whereas the maximum difference in video-based locomotion activity ranged between 18.58 and 23.42 m, depending on the minimum movement threshold (black vs. pink color-marked broiler).

**Table 3:** Total distance moved (m) per color-marked broiler as derived from radio frequency identification (RFID) and video.

		Video			
Broiler	RFID	as RFID	Pixel-based	Pixel-based (>5 mm)	Pixel-based (>5 cm)
Black	24.10	74.99	59.79	46.46	19.97
Light blue	24.79	63.68	74.67	61.91	31.41
Pink	27.85	94.42	82.71	69.88	38.55

Locomotion activity derived from video presented as RFID, including the defective antenna, and as pixel-based with minimum movement thresholds of 5 mm and 5 cm.

#### Apparent Feeding and Drinking Behavior

Apparent feeding behavior showed large discrepancies in the number of visits and the total duration of the visits between both systems (Table 4). Compared to RFID, the number of apparent feeding visits was approximately 3 to 6 times higher when using video-as-RFID, and approximately 2 to 3 times higher when using video pixel-based. A similar pattern can be seen for the total duration of the visits for two of the birds, where video-as-RFID and video pixel-based were approximately 2 to 3 times higher compared to the RFID-system. When using the RFID-system, the black color-marked broiler had the fewest visits but the longest total duration of feeder visits (even longer than on video; Table 4). The number of apparent feeding visits was approximately 2 to 3 times higher for video-as-RFID compared to video pixel-based, but the total duration of those visits was similar.

**Table 4:** Apparent feeding behavior of each color-marked broiler in number of visits and total duration (seconds) as derived from radio frequency identification (RFID) and video.

	RFID		Video			
			as RFID		Pixel-based	
Broiler	Number of visits (#)	Total duration (sec)	Number of visits (#)	Total duration (sec)	Number of visits (#)	Total duration (sec)
Black	6	1022	33	447	11	488
Light blue	8	288	44	759	24	882
Pink	10	372	34	653	24	594

Video presented as video-as-RFID and pixel-based.

Similar observations were made for the apparent drinking behavior. Apparent drinking behavior showed large discrepancies in the number of visits and the total duration of the visits between both systems. The number of apparent drinking visits were approximately 3 to 6 times higher when using video-as-RFID than when using the RFID-system. When using the RFID-system, the pink color-marked had the shortest total duration of drinker visits, but the longest total duration when using video-as-RFID (Table 5).

**Table 5:** Apparent drinking behavior of each color-marked broiler in number of visits and total duration (seconds) as derived from radio frequency identification (RFID) and video.

	RFID		Video	
Broiler	Number of visits (#)	Total duration (sec)	Number of visits (#)	Total duration (sec)
Black	2	291	7	355
Light blue	5	100	14	359
Pink	3	60	17	436

Video presented as video-as-RFID.

## DISCUSSION

In this study a comparison was made between RFID and video for the determination of location and movement in broilers. The potential of both systems to assess individual behavioral metrics, such as space usage, locomotion activity and apparent feeding and drinking behavior was shown. One main advantage of our study was the opportunity to study two sensor systems simultaneously, under the same circumstances. Moreover, we were able to improve our understanding of the application of RFID and video to derive individual broiler behavioral metrics.

### Localization Agreement

A comparison between the RFID-system derived and video-based animal locations was made to assess the localization agreement between both systems. The localization agreement was based on the RFID-system derived and video-based the trajectories of the birds. Differences between RFID-derived and video-based animal locations were expected, given that the RFID-antenna's center point was used as animal location and the animal is unlikely to be in the exact center of an antenna. Hence, the percentage of differences less than or equal to the mean radius of the antennas’ enclosing circle was used as a measure of agreement. Most of the animal location differences (77.5%) occurred within the mean radius of the antennas’ enclosing circle (≤128 px, 28.15 cm), and 95.3% of the differences were within a one antenna difference (≤256 px, 56.30 cm). It is important to note that these differences can originate from both systems and relate to differences in location determination and movement detection.

### Location Determination

The location reference point to determine the location of the animal differed between the two systems. The RFID-system derived animal location was based on the position of the RFID-tag attached to one of the broilers’ legs, whereas the video-based location was based on the detected bounding box's center point. As a result, the location reference point of the two systems could be located at different antennas in some cases (van der Sluis et al., 2020). In other words, a broiler near the edge of two antennas repositioning its leg could have been recorded as a change in location for the RFID-system, whereas the actual movement of the broiler was minimal. This does not necessarily mean that the RFID-system is sensitive to the broiler's leg repositioning near antenna edges because the RFID-tag is not always detected. Similarly, minimal movement of the detected bounding box's center point near antenna edges could easily cause a switch between antennas for video-as-RFID, which is reflected by the high number of switches recorded (Appendix Figure A-1). The animal's behavior, changes in posture while lying down, and detection noise all influenced where the detected bounding box's center was located and hence to which antenna it was assigned for video-as-RFID (Doornweerd et al., 2022).

### Movement Detection

The presence on or movement over an antenna for the broilers was not always registered by the RFID-system. On video, movement to antennas non-adjacent to the previously recorded antenna was observed only for the light blue color-marked broiler on video. Consecutive RFID-readings or detections on video-as-RFID could comprise non-adjacent antennas, and differences larger than two antennas could be observed, as well.

Van der Sluis et al. (2020) provide two possible explanations for the missed registrations of the RFID-system, tag orientation and a broiler's velocity. Redfern et al. (2017) showed that a tag orientation perpendicular to the antenna worked best to register an RFID-tag, but that registration was dependent on tag orientation in relation to the magnetic field of the RFID-antenna. The velocity of a broiler may also have had an effect on the registration of the RFID-tag. Gebhardt-Henrich et al. (2014) tested the success rate of tag registration for 5 different tag orientations and 5 different tag velocities (0.5–3.0 m/s, increments of 0.5 m/s) for a low-frequency RFID-system. In that study, faster movement of an RFID-tag over the antenna decreased the probability of being registered. They also found an interaction between tag orientation and velocity of an RFID-tag. A tag orientation parallel to the antenna had the highest probability of being registered and showed the smallest decline in probability of registration with increasing velocities. However, both Gebhardt-Henrich et al. (2014) and Redfern et al. (2017) used a low-frequency RFID-system in contrast to the high-frequency RFID-system of this study (van der Sluis et al., 2020). According to Gebhardt-Henrich et al. (2014), the decline in registration probability is the sharpest for speeds over 1.5 m/s. In broilers, different speeds (mean ± SD) for different body weights have been reported from 0.34±0.17m/s (3.5±0.3kg,n=9; Paxton et al., 2013) to 1.01±0.38m/s (1.84±0.13kg,n=10; Caplen et al., 2012). In the study of Caplen et al. (2012), the velocities were measured after a period of feed withdrawal. Hence similar speeds are less likely to occur in this study. Nonetheless, van der Sluis et al. (2020) made similar observations and noted that movement of broilers that do not fully rise while moving may not have been registered, that is, the tag orientation in relation to the magnetic field of the RFID-antenna was inadequate.

The presence on or movement over an antenna for the light blue color-marked broiler was not always registered on video. In this study, the detection on video could switch between the light blue and dark blue color-marked broilers. Two consecutive nonoverlapping detections indicated a possible switch between the animals, which was corrected by assuming that the animal remained at its last known location. Three consecutive nonoverlapping detections could be potential movement, which was not corrected. This correction strategy worked for short switches, but not for longer switches. The light blue color-marked broiler was still observed to move to nonadjacent antennas, which may be attributed to non-corrected detection switches between the light blue and dark blue color-marked broiler. In this study, filtering and thresholding were kept to a minimum with the assumption that the animal remained at its last known location in case of missing detections on video or missing reads by the RFID-system. The reduction of video detections from 25 frames per seconds to 1 frame per second inadvertently applied a filter that reduced the number of switches between objects. Unreduced detections showed switches between some color-marked birds (light blue and dark blue, black and dark blue), as well as the black color-marked broiler and the black top of the drinker. The occurrence of these switches highlights the importance of a good detection model, especially when moving to higher frame rates. The animal's behavior, changes in posture while lying down, and detection noise all influenced where the detected bounding box's center was located, and consequently, video is sensitive to movement of the bounding box's center point which might not necessarily be movement of the animal as such (Appendix Figure A-1).

### Behavioral Metrics

Differences between the systems in the recorded values of the behavioral metrics stem from the way that location is determined and the sensitivity of the system to changes in location. The systems’ agreement on animal location indicates that both systems should report similar approximations of space usage and apparent feeding and drinking behavior. However, the ability of a system to detect movement is critical, especially when concerned with locomotion activity. A system's method of location determination as well as its sensitivity to changes in location are related and will thus be discussed in parallel when discussing the behavioral metrics.

The RFID-system and video both showed a similar general area pattern in which the animal spent the most time. However, a general area pattern does not suffice for the determination of apparent feeding and drinking behavior, where the time spent and number of visits to a specific area are important. The definition of that specific area plays an important role. In this study, the feeder areas were defined by the RFID-antenna or a circular sector, and the drinker by the RFID-antenna. An animal's presence on a feeder or drinker associated RFID-antenna does not equate actual feeding or drinking behavior, which is why we define it as apparent feeding and drinking behavior. Apparent feeding and drinking behavior could be used as a proxy for actual feeding or drinking behavior. Actual feeding behavior is more uncertain than drinking behavior given the ratio of the RFID-antenna area to the area in which the animal can reach the feed.

Broiler movement on or off an antenna was not always registered by the RFID-system, and with the assumption that the animal remained at its last known location this could result in missed visits and overestimated or underestimated time-spent on antennas (Appendix Figure A-1). The moist bedding likely affected the antenna under the drinker, causing it to miss or delay the animal's registration on the antenna (van der Sluis et al., 2020). Video-based space usage and apparent feeding and drinking behavior were affected by small movements of the detected bounding box's center point, and hence the antenna to which it was assigned. This increased the number of antenna switches and thus the number of visits on feeder and drinker antennas but did not necessarily have a big influence on the recorded total time spent. When the feeder area was defined as a circular sector, the number of feeder visits decreased, but time spent remained largely unchanged compared to the situation in which the RFID-antenna was used as the feeder area.

Likewise, differences in locomotion activity between the two systems could be explained by similar mechanisms. The RFID-system did not always register broiler movement on or off an antenna which could result in movement being missed, whereas video-based animal locations were sensitive to small movements of the detected bounding box's center point. Moreover, movement could only be registered between antennas for the RFID-system, whereas video could register movement within and between antennas. The effect of the small movements of the bounding box's center point was reduced with a minimum movement threshold. However, there was still a difference in locomotion activity within-broilers between RFID and video pixel-based. This locomotion activity difference is potentially movement within antennas but revealed a likely overestimation of movement by RFID for the black color-marked broiler (Table 3).

Overall, behavioral metrics from video are most probably closer to the true behavioral metrics than those from the RFID-system. The RFID-system and video gave similar indications of space usage, and similar animal rankings for locomotion activity. However, video provides more detail than the RFID-system and had more pronounced differences in locomotion activity. In this experiment, video seemed better suited than the RFID-system for determining apparent feeding and drinking behavior than the RFID-system.

### Prospects

Broiler location, movement, and behavioral metrics are likely best derived from video. The RFID-system gives good approximations of broiler location, movement, and behavioral metrics, except for apparent feeding and drinking behavior, but has limitations to the level of detail it can provide. Nevertheless, at present, only the RFID-system can provide individual identification for non-color marked broilers. Potentially, a sensor-fusion approach can combine the strengths of video and an RFID-system (Ellen et al., 2019) in a combination of verifiable and detailed video with the unique identification of RFID. The proposed combination has been implemented for different livestock species in different settings (e.g., Nakarmi et al., 2014; Guzhva et al., 2018 and van Hertem et al., 2018) and seems promising. However, implementation will require detection and tracking of non-color marked broilers on video in commercial environments under varying conditions (e.g., group size, stocking density, lighting, background), algorithm development to link detected and registered animals, and a study on the placement and number of required RFID-antennas.

The details in a video make it possible to identify, describe, and quantify a wide range of behaviors. The potentially wide range of recorded behaviors of specific broilers and its conspecifics offers the opportunity to place the behavior into context. Individual broiler behavioral data presents opportunities to improve the precision of breeding, feeding, and management (Koltes et al., 2019). Often new techniques are not implemented in practice due to being impractical, complex, or expensive (Koltes et al., 2019). Impracticality, complexity, and expenses arise from the required hardware, software and integratability in the husbandry system (e.g., data migration, data storage; Neethirajan, 2020). However, the benefits of individual behavioral data could outweigh the costs (Wurtz et al., 2019). Ultimately, the choice for a sensor system depends on the objective and available resources.

## CONCLUSION

In this study a comparison was made between RFID and video for the determination of location and movement in broilers. The potential of both systems to assess individual behavioral metrics, such as space usage, locomotion activity and apparent feeding and drinking behavior, was shown. Animal locations derived from the RFID-system and based on video were largely in agreement. Most animal location differences (77.5%) were within the mean radius of the antennas’ enclosing circle (≤128 px, 28.15 cm), and 95.3% of the differences were within a one antenna difference (≤256 px, 56.30 cm). Animal movement was not always registered by the RFID-system whereas video was sensitive to detection noise and the animal's behavior (e.g., pecking). The method used to determine location and the systems’ sensitivities to movement led to differences between the obtained behavioral metrics. The RFID-system gives good approximations of broiler location, movement and behavioral metrics, with the exception of apparent feeding and drinking behavior, but has limitations in the level of detail it can provide. Broiler location, movement, and behavioral metrics are likely best derived from video. Currently, only the RFID-system can provide individual identification for non-color marked broilers. Potentially, a combination of verifiable and detailed video with the unique identification of RFID will make it possible to identify, describe, and quantify a wide range of individual broiler behaviors. Individual broiler behavioral data presents opportunities to improve the precision of breeding, feeding, and management.

## Figures and Tables

**Table A-1:** Number of visited antennas, total difference time spent between radio frequency identification (RFID) and video (mean ± SD) and number of switches as derived from video-as-RFID excluding defective antenna.

Broiler	Number of unique visited antennas (#)	Difference total time spent per antenna (sec)	Number of switches between antennas (#)
Black	26	0.00 ± 302.15	193
Light blue	17	0.00 ± 179.13	157
Pink	25	0.00 ± 375.56	223

**Table A-2:** Total distance moved (m) per broiler as derived from radio frequency identification (RFID) and video-as-RFID excluding defective antenna.

	RFID	Video-as-RFID
Broiler		
Black	24.10	73.92
Light blue	24.79	63.68
Pink	27.85	90.46

## Appendix B Supplementary materials

Image, application 1
