# Supplementary material for: Passive radio frequency identification and video tracking for the determination of location and movement of broilers
Source: Poult Sci. 2022 Dec 9;102(3):102412. doi: 10.1016/j.psj.2022.102412 (PMC9841275; doi:10.1016/j.psj.2022.102412)
Supplement: Supplementary file 1 [file mmc1.docx]

**Supplementary Information**

**Passive radio frequency identification and video tracking for the determination of location and movement of broilers.**

**J.E. Doornweerd*^1^, G. Kootstra^†^, R.F. Veerkamp*,** **B. de Klerk^‡^, I. Fodor*, M. van der Sluis*, A.C. Bouwman* and E.D. Ellen***

^*^ Animal Breeding and Genomics, Wageningen University & Research, 6700AH Wageningen, the Netherlands
^†^ Farm Technology, Wageningen University & Research, 6700 AA Wageningen, the Netherlands,
^‡^ Research & Development, Cobb Europe BV, 5831 GH Boxmeer, the Netherlands
^1^ [janerik.doornweerd@wur.nl](mailto:janerik.doornweerd@wur.nl)
Correspondence address: Wageningen Campus, Building 107, Droevendaalsesteeg 1, 6708 PB, Wageningen, the Netherlands

**Hyperparameters**

# YOLOv5 ðŸš€ by Ultralytics, GPL-3.0 license

# Hyperparameters for COCO training from scratch

# python train.py --batch 40 --cfg yolov5m.yaml --weights '' --data coco.yaml --img 640 --epochs 300

# See tutorials for hyperparameter evolution https://github.com/ultralytics/yolov5#tutorials

lr0: 0.01 # initial learning rate (SGD=1E-2, Adam=1E-3)

lrf: 0.2 # final OneCycleLR learning rate (lr0 * lrf)

momentum: 0.937 # SGD momentum/Adam beta1

weight_decay: 0.0005 # optimizer weight decay 5e-4

warmup_epochs: 3.0 # warmup epochs (fractions ok)

warmup_momentum: 0.8 # warmup initial momentum

warmup_bias_lr: 0.1 # warmup initial bias lr

box: 0.05 # box loss gain

cls: 0.5 # cls loss gain

cls_pw: 1.0 # cls BCELoss positive_weight

obj: 1.0 # obj loss gain (scale with pixels)

obj_pw: 1.0 # obj BCELoss positive_weight

iou_t: 0.20 # IoU training threshold

anchor_t: 4.0 # anchor-multiple threshold

# anchors: 3 # anchors per output layer (0 to ignore)

fl_gamma: 0.0 # focal loss gamma (efficientDet default gamma=1.5)

hsv_h: 0.015 # image HSV-Hue augmentation (fraction)

hsv_s: 0.7 # image HSV-Saturation augmentation (fraction)

hsv_v: 0.4 # image HSV-Value augmentation (fraction)

degrees: 0.0 # image rotation (+/- deg)

translate: 0.1 # image translation (+/- fraction)

scale: 0.5 # image scale (+/- gain)

shear: 0.0 # image shear (+/- deg)

perspective: 0.0 # image perspective (+/- fraction), range 0-0.001

flipud: 0.0 # image flip up-down (probability)

fliplr: 0.5 # image flip left-right (probability)

mosaic: 1.0 # image mosaic (probability)

mixup: 0.0 # image mixup (probability)

copy_paste: 0.0 # segment copy-paste (probability)


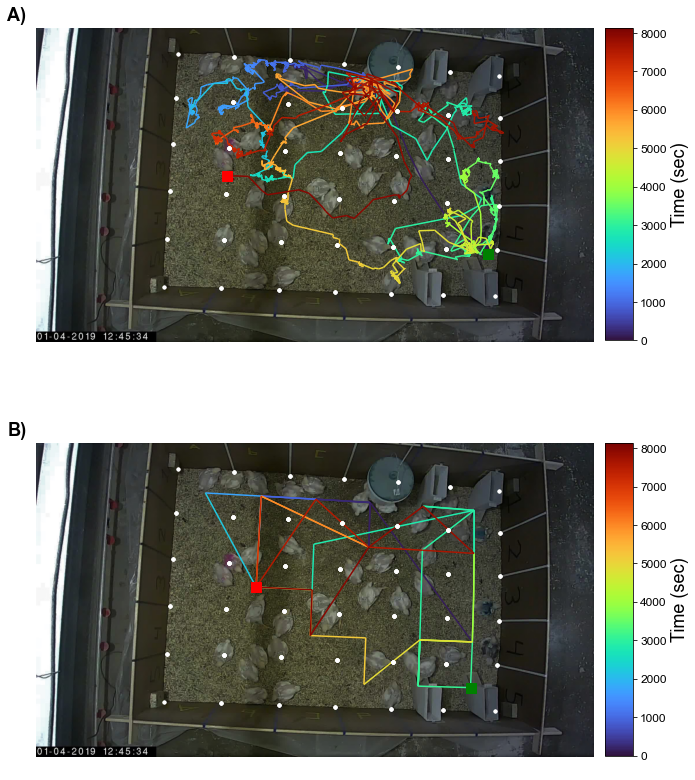


Figure S1. Location of the pink color-marked broiler over time as derived from video (A) and radio frequency identification (RFID; B). The green square represents the track’s start, and the red square represents the track’s end. The color transition depicts the location’s progression from dark blue to dark red over time. The white dots represent the antenna corners. The plot is projected over a darkened video freeze frame.


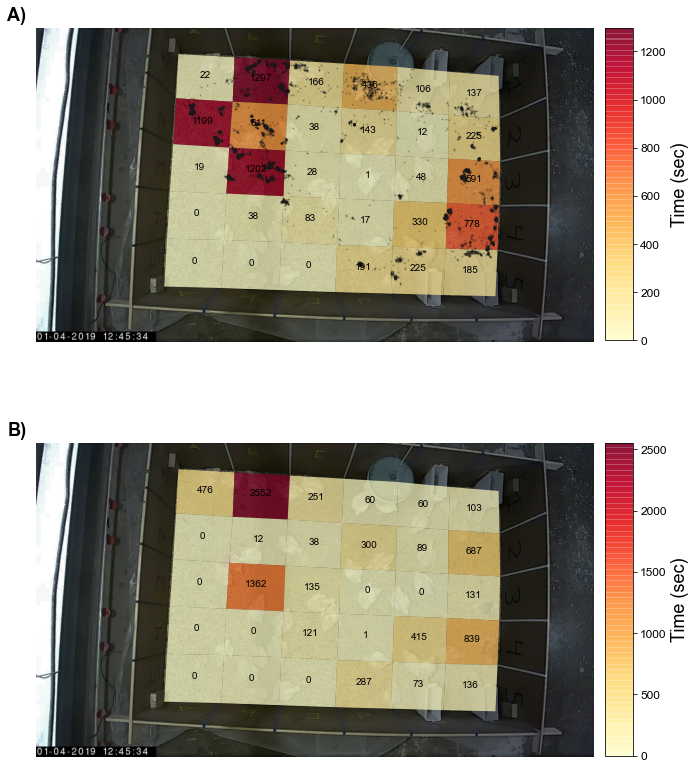


Figure S2. Heatmaps of the pink color-marked broiler as derived from video (A) and radio frequency identification (RFID; B). The numbers represent the total amount of time spent on each antenna in seconds. RFID antenna A2 was defective. The animal’s bounding box center point locations are included in the heatmap derived from video locations (A) as black dots. The plot is projected over a darkened video freeze frame.


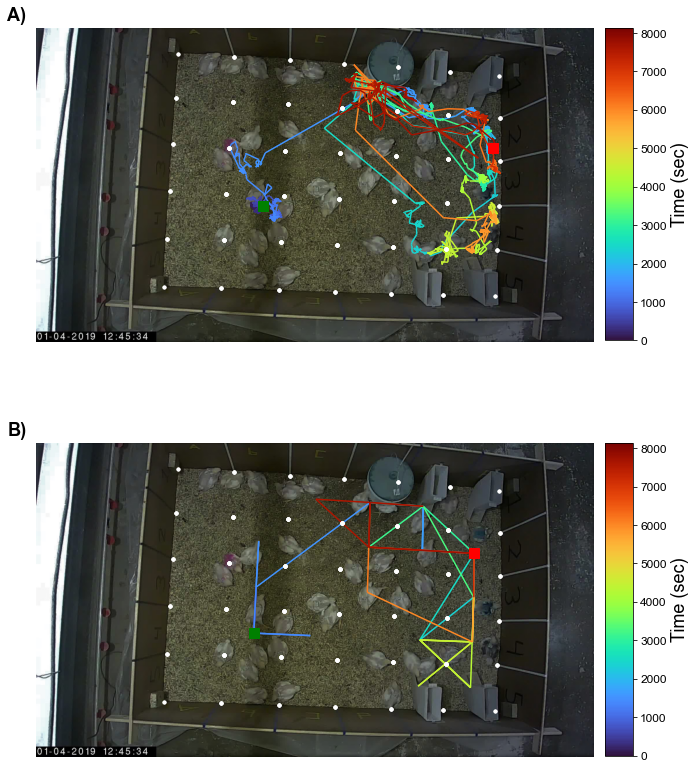


Figure S3. Location of the light blue color-marked broiler over time as derived from video (A) and radio frequency identification (RFID; B). The green square represents the track’s start, and the red square represents the track’s end. The color transition depicts the location’s progression from dark blue to dark red over time. The white dots represent the antenna corners. The plot is projected over a darkened video freeze frame.


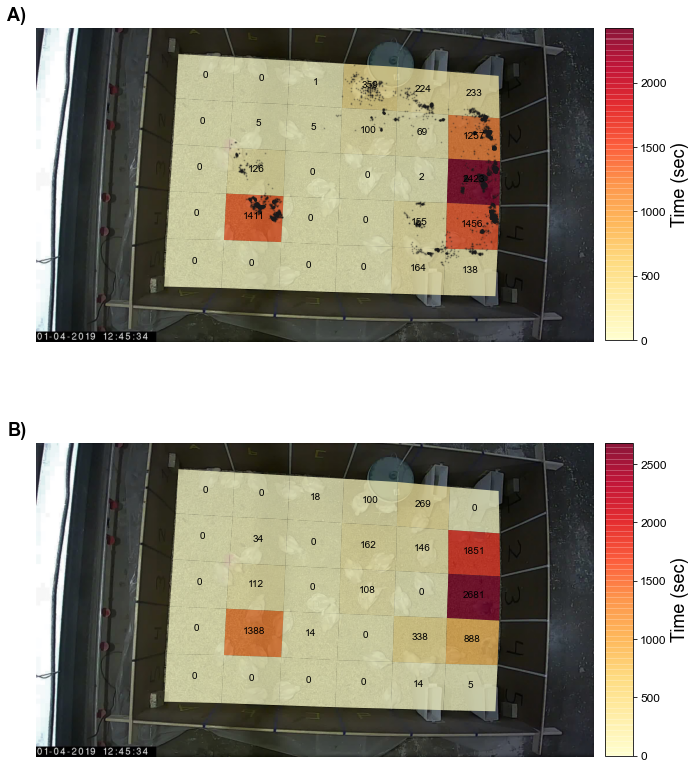


Figure S4. Heatmaps of the light blue color-marked broiler as derived from video (A) and radio frequency identification (RFID; B). The numbers represent the total amount of time spent on each antenna in seconds. RFID antenna A2 was defective. The animal’s bounding box center point locations are included in the heatmap derived from video locations (A) as black dots. The plot is projected over a darkened video freeze frame.
